# Advances in the development of an imaging device for plaque measurement in the area of the carotid artery

**DOI:** 10.1080/13102818.2014.910362

**Published:** 2014-07-08

**Authors:** Lačezar Ličev, Michal Krumnikl, Jaromír Škuta, Marek Babiuch, Radim Farana

**Affiliations:** ^a^ Faculty of Electrical Engineering and Computer Science, VŠB-Technical University of Ostrava, Ostrava-Poruba, Czech Republic; ^b^ Faculty of Mechanical Engineering, VŠB-Technical University of Ostrava, Ostrava-Poruba, Czech Republic

**Keywords:** ultrasound probe, plaque measurement, linear movement, ECG synchronization

## Abstract

This paper describes the advances in the development and subsequent testing of an imaging device for three-dimensional ultrasound measurement of atherosclerotic plaque in the carotid artery. The embolization from the atherosclerotic carotid plaque is one of the most common causes of ischemic stroke and, therefore, we consider the measurement of the plaque as extremely important. The paper describes the proposed hardware for enhancing the standard ultrasonic probe to provide a possibility of accurate probe positioning and synchronization with the cardiac activity, allowing the precise plaque measurements that were impossible with the standard equipment. The synchronization signal is derived from the output signal of the patient monitor (electrocardiogram (ECG)), processed by a microcontroller-based system, generating the control commands for the linear motion moving the probe. The controlling algorithm synchronizes the movement with the ECG waveform to obtain clear images not disturbed by the heart activity.

## Introduction

Cardiovascular diseases are one of the top leading causes of death. From this category, ischemic stroke is one of the most frequent causes. Ischemic stroke, the most common type of stroke, results from blockage of a blood vessel carrying oxygen to the brain. Therefore, the detection of beginning atherosclerotic disease is extremely important for prevention of acute events.

Conventional two-dimensional (2D) ultrasound imaging is usually an adequate and cost effective method for evaluation of composition and surface features of plaque. However, this method does not provide necessary information about the total plaque volume and its development in time.

A variety of other imaging techniques have been proposed for monitoring and detection of atherosclerotic plagues in recent years. These techniques range from magnetic resonance (MR) imaging,[1,2] optical coherence tomography,[3] high-frequency duplex ultrasound,[4] intravascular ultrasound,[5] to coronary angioscopy. Nevertheless, measurements using ultrasonography or angiography remain the principal method for detection of atherosclerotic plagues and determination of the severity of carotid atherosclerosis.

High-spatial-resolution MR is currently described as one of the most promising imaging techniques.[6,7] A review of a high-spatial-resolution MR using a widely available 1.5-T clinical imager and phased-array surface coil was published by Yuan et al.[6] MR images can be further analysed using e.g. active shape models [8,9] or segmentation techniques.[10,11]

In this paper, we propose an imaging technique based on precise movement of the ultrasonic probe. The images obtained from each slice are processed to create a 3D model of the scanned blood vessel. The advantage of this approach is usage of a standard, widely available ultrasonic probe and a relatively cheap and easily accessible positioning device.

A similar approach has already been successfully tested on animals, e.g. the 3D ultrasonic reconstruction from freehand 2D interrogations, using the standard ultrasonic probe described by Allott et al.[11] The studies showed that ultrasound is able to detect and quantify a low echo structure corresponding to fatty streaks in the Watanabe rabbit aortic arch. In Chiu et al.,[12] intima-media thickness was measured by means of 2D ultrasound images extended to a 3D model. The distance between the carotid wall and lumen surfaces was established on a point-by-point basis. A comparison between the spatial compound ultrasound models and anatomical models of carotid atherosclerotic plaque was also published.[13]

In this paper, we describe a system for 3D ultrasound measurement based on the model created from the series of 2D images. The precise positioning system is introduced; its hardware parts and necessary software for probe movement and electrocardiogram (ECG) synchronizations are described; and results from clinical studies are provided.

The system works on the principle of merging the traditional 2D images captured by ultrasound: individual cross sections depicting the carotid artery including the plaque. By combining these objects from the individual images, we create an overall 3D model of arteries and plaque. Diagnostic parameters such as the total volume of the artery, plaque volume, etc., are calculated from the obtained model.

We have implemented a mechanism for creating 3D images of the artery, based on a precision movement of the ultrasound probe. The probe allows displaying cross-sections (2D) at defined distances. It should be noted here that the pulse of the heart affects the shape of the scanned section (2D artery cross-section). For this reason, it is necessary to synchronize the image capturing of the cross-section with the heartbeat. The goal was to create a control chain (hardware equipment and software tools), which allows the synchronization of the ultrasonic probe with the heartbeat.

## Materials and methods

### Positioning system of ultrasound probe

**Figure 1.**
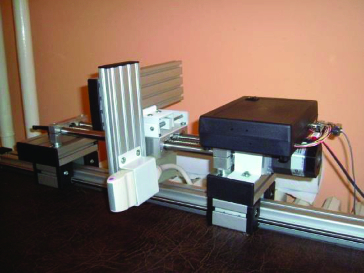
Positioning mechanism of the ultrasound probe.

For a description (also terminology) and explanation of how we create mechanisms, we use an analogy of some parts from lathe construction. The images were obtained using a GE VIVID 7 Pro ultrasound device. The positioning device is composed of components made by ULMER. The base part is fixed to the patient's bed (see Figure 1). On that bed, the base can be moved manually with a longitudinal mechanism, which allows the examining physician to set the position of the ultrasound probe according to the patient's position. The position of the probe relative to the patient's neck can be adjusted through a mechanism that moves the probe in the transverse direction. The ultrasonic sensor is located on a shift platform which is implemented using a stepper engine from MICROCON. Movement of the probe is within the range of 2–3 cm.

### Triggered signal generator – patient monitor

The ECG signal is used for the synchronization of the probe movement control. The ECG signal sensing is done with electrodes that are connected to the recording instrument. The electrodes are placed on the skin of the four limbs and the chest. The first wave of the signal which can be seen on the ECG is the P-wave, which suggests starting of a contraction. It is not possible to recognize the ventricular repolarization on the ECG as a relevant biosignal, since it is overshadowed by a much stronger signal originating from the ventricular depolarization[14]; this signal is characterized by a QRS complex wave. The following T-wave indicates the other ventricular repolarization.[14]

**Figure 2.**
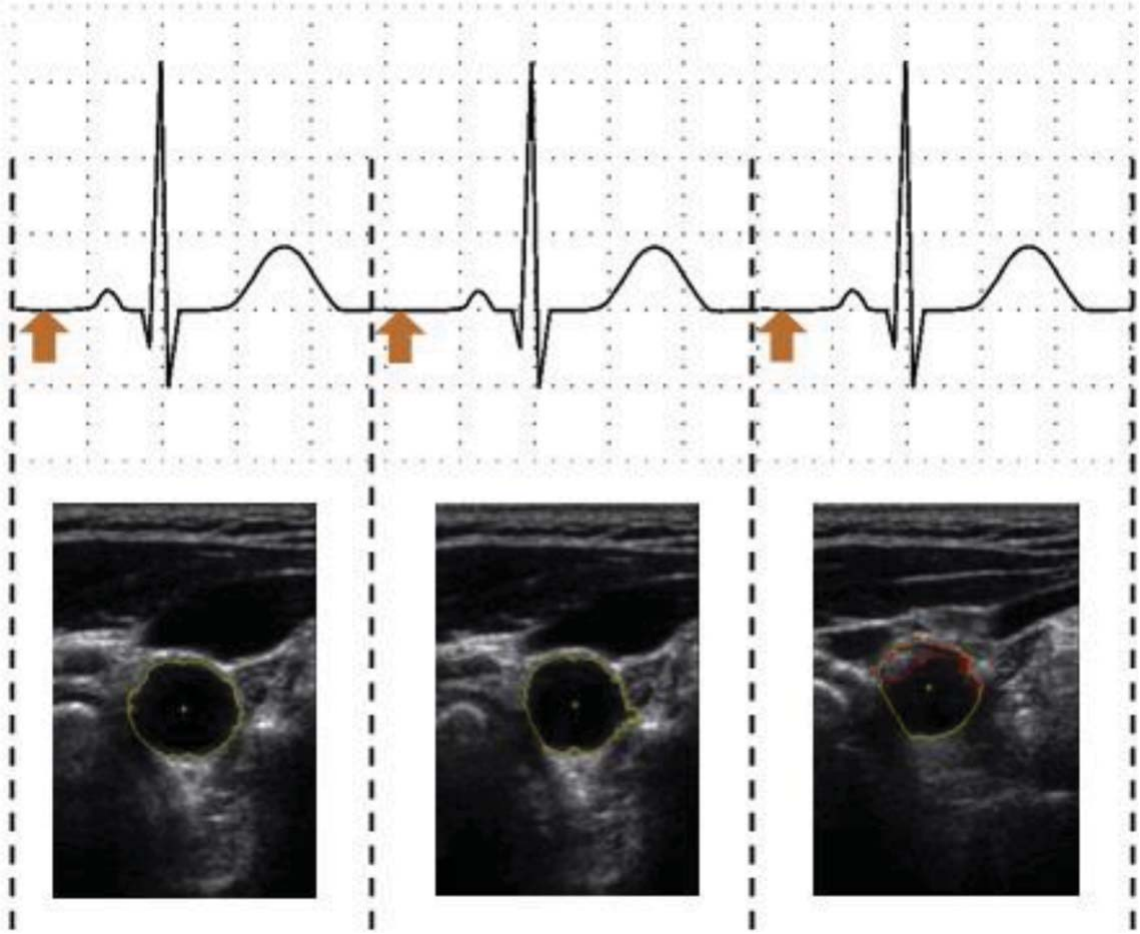
ECG signals with highlighted trigger points.

Figure 2 shows the ECG waveform with a gap in the middle. The space between the heartbeats is currently used for capturing the image. The experiments were performed on a multipurpose portable patient monitor (S5 Compact Anaesthesia Monitor), which is used to monitor ECG, heart rate, temperature, etc. The amplified output of the ECG signal (1 V/1 mV) is used to synchronize the movement of the ultrasonic probe. The sample of the processed data is given as online supplemental content available for download.

As a stepper motor we used an SM2321-1400 motor (static torque of 1.5 Nm, 200 steps per revolution) and a threaded-rod with 5 mm rise for a thread. From these parameters we can see that during the single step of the stepper engine, a shift of 0.025 mm is achieved. In terms of positioning accuracy of the ultrasonic probe (considering the will of the thread), we have achieved accuracy of 0.5 mm.

The measurements were performed with the following parameters: a sliding path of 25 mm (forward/reverse), while the time interval of the ultrasound probe shift was chosen with regard to the patient's heart rate and carefully examined by physicians to trigger the signal correctly according the ECG signal.[14]

The system is based on popular ATmega128 microcontroller.[15] The controller already contains an A/D input; therefore, we do not need to use an external A/D converter for the triggering signal. When the input value exceeds the given threshold set in the controlling software, the unit generates all commands for the power unit and the DC30M stepper motor control unit. The system includes an RS-232 interface for the communication with the control and power unit.

**Figure 3.**
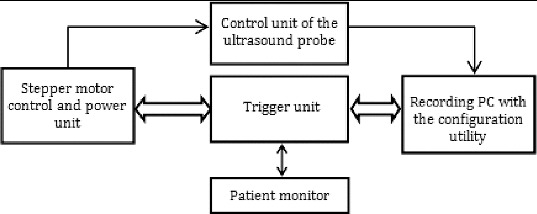
Block diagram of the system.

Figure 3 shows a block diagram of the proposed control system. The figure depicts the logical division of functions among the system parts.

### Application software

The application software is divided into several logical levels. The first level is a programme support for the triggering unit. The algorithm for triggering and correct displacement of the ultrasonic probe is implemented in the microcontroller firmware. The operator's computer connected to the device runs a monitoring utility. The main functions are to configure and monitor the time periods of the triggering signals. For a subsequent off-line evaluation, the time stamps are sent over the serial interface to record all events of the control unit. The timestamps are later used to define the time periods of the artery cut at the time of its release. The system is automatically adapting to the changes of the patient's heart rate.

## Results and discussion

### Applications in clinical studies

The clinical studies using this device were performed at the Faculty Hospital Ostrava, Department of Neurology and Faculty of Medicine, Ostrava University. The full study was published in Bar et al.[16]

The aim of the study was to determine the conformity of the evaluation done by the two ultrasonographists on the following parameters: echogenicity, homogeneity, surface volume and maximum content of plaque. The edges of the plaque in each image were manually identified by the investigators. The total volume of plaque was calculated upon the transformation of 2D images into 3D format, using the FOTOM application.

The inter-observer agreement for echogenicity, homogeneity and surface was assessed using weighted kappa coefficient. The agreement was considered poor when the kappa was <0.4, good 0.41–0.75 and excellent >0.75. The parametric values (content and volume) were tested by a paired *t*-test. The value of *P* ≤ 0.05 was considered significant for the disagreement.

The study enrolled 30 patients (mean age 72 ± 13 years) with 28 evaluated atherosclerotic plaques. Inter-rater agreement for homogeneity was 96% (kappa = 0.84, *P* < 0.001); for surface 90% (kappa = 0.77, *P* < 0.001); and for echogenicity 86% (kappa = 0.60, *P* < 0.001). The agreement for measurement of plaque content and volume was kappa = 0.31 and 0.30, respectively, and the correlation coefficient was 0.808. The full medical statistics and results are published in Bar et al.[16]

While the number of evaluated plaques was relatively small, it was sufficient for statistical analysis. The inter-rater agreement of 2D and 3D ultrasound evaluation of atherosclerotic plaque parameters was excellent.

**Figure 4.**
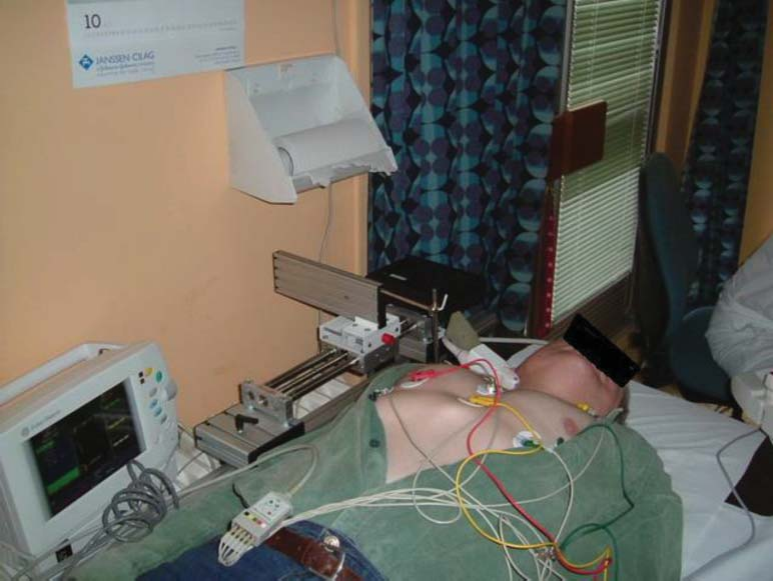
Configuration of the proposed device for the 3D ultrasound display and measurement of the atherosclerotic plaque.

**Figure 5.**
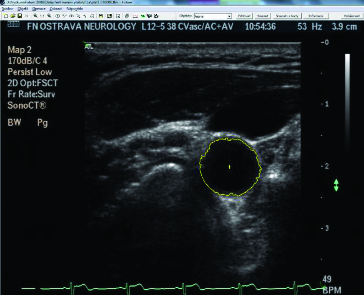
Example of the cross-section of the artery with highlighted object inside the measuring application.

Figure 4 shows the patient examination by the proposed device. Figure 5 depicts a snapshot of the cross-section of carotid arteries taken by the device.

The proposed device was described as simple, quick and cost effective for screening. The measurements of the total plaque volume were most accurate and, therefore, the device was considered to be used for the risk-assessment of plaques.[16]

## Conclusions

This paper describes a novel method for measuring the atherosclerotic plagues using ultrasound. The proposed solution was used during a clinical study and compared with independent evaluations performed by two physicians. The results were correlated and found in accordance with the reference data. The main contribution of this approach is the possibility of using already available equipment without the need of expensive MR devices. In this way, proper diagnostics is available to a wider range of patients. Additionally, the diagnostics can be performed much faster, without the unnecessary delays for MR. The disadvantage of the current state is the need for manual outlining of the plaque boundaries and our future work is aimed at investigating the accuracy of an automatic or semiautomatic system for plaque boundary detection and comparison with the gold standard for non-invasive imaging of atherosclerotic plagues: MR imaging. 

## Supplemental data

Supplemental data for this article can be accessed at http://www.cs.vsb.cz/licev/experiments/test2.zip.
